# Enhancement of Frequency Stability Using Synchronization of a Cantilever Array for MEMS-Based Sensors

**DOI:** 10.3390/s16101690

**Published:** 2016-10-13

**Authors:** Francesc Torres, Arantxa Uranga, Martí Riverola, Guillermo Sobreviela, Núria Barniol

**Affiliations:** Electrical Engineering Department, Universitat Autònoma de Barcelona, Edifici Q, Campus UAB Bellaterra, Cerdanyola del Vallès 08193, Spain; Arantxa.Uranga@uab.cat (A.U.); Martin.Riverola@uab.cat (M.R.); Guillermo.Sobreviela@uab.cat (G.S.); Nuria.Barniol@uab.cat (N.B.)

**Keywords:** MEMS, synchronization, resonators, CMOS-MEMS, cantilevers, arrays, coupling

## Abstract

Micro and nano electromechanical resonators have been widely used as single or multiple-mass detection sensors. Smaller devices with higher resonance frequencies and lower masses offer higher mass responsivities but suffer from lower frequency stability. Synchronization phenomena in multiple MEMS resonators have become an important issue because they allow frequency stability improvement, thereby preserving mass responsivity. The authors present an array of five cantilevers (CMOS-MEMS system) that are forced to vibrate synchronously to enhance their frequency stability. The frequency stability has been determined in closed-loop configuration for long periods of time by calculating the Allan deviation. An Allan deviation of 0.013 ppm (@ 1 s averaging time) for a 1 MHz cantilever array MEMS system was obtained at the synchronized mode, which represents a 23-fold improvement in comparison with the non-synchronized operation mode (0.3 ppm).

## 1. Introduction

Nano and micro electromechanical systems (M/NEMS) have been widely used for single or multiple mass detection due to their small effective masses and high resonance frequencies. Ultimate limits in mass sensing have been reported with NEMS-based resonators [1]. In dynamic or resonant mode, one of the main characteristics that limits the mass resolution is the stability of the resonance frequency of the M/NEMS resonator. This limitation comes from multiple sources of noise, which become more important with the size reduction to the nanoscale of the NEMS devices as has been recently reported [2]. 

Synchronization is described as an adjustment of rhythms of oscillating objects due to their weak interaction [3]. From the first work of Huygens [4] until the present day, there has been great effort to provide a comprehensive theoretical basis to synchronization [5] and, derived from studies involving collective systems, mathematical techniques to extract from the data the pure synchronous behavior from heterodox phenomena [6]. Recently, synchronization phenomena have become an interesting issue in a wide range of scientific fields [7,8,9]. In the field of M/NEMS, synchronization phenomena are rising in importance and a lot of scientific works have appeared using a few synchronized elements [10], a lot of them [8], or using synchronization to enhance the mass detection performance of MEMS-based sensors [11].

Special efforts have focused on studying synchronization based on different methods of interaction between MEMS and its influence on frequency stability [12]. There are important works related to this phenomenon dealing with mechanical interactions [11] or electrical interaction between MEMS [13,14], new sensing strategies like localized modes [15], or stiffness change [16]. In addition, there is a parallel effort to enhance the sensors’ performance, trying to use non-linear phenomena to overcome noise limitations [17,18,19].

We have focused our study on trying to take advantage of a synchronized MEMS system in a closed-loop oscillator to enhance the frequency stability over time using a fully integrated CMOS system, with electrical and mechanical interaction between resonating elements. An example of the utility of synchronization is that it allows for obtaining a higher performance single or multiple mass sensor. 

This paper is organized as follows: Section 2 describes the design of the system (based on five cantilevers that are mechanically connected), the fabrication method (based on CMOS technology), and the electrical characterization of the system. In this Section 2, the modal frequencies of the five cantilevers (obtained in an open-loop configuration), as well as the frequency at which the system vibrates at closed-loop configuration with its stability in time, are provided. From these results the mass sensitivity of the system (in a closed-loop configuration) is computed. Section 3 is devoted to characterizing the stability of the frequency in time in a closed-loop configuration when the system is forced to synchronously vibrate using an external force. The authors present two different ways to force the synchronization, the first one acting over one individual cantilever at its modal frequency and the other one acting over the same cantilever but at the frequency at which the system vibrates in a closed-loop configuration. In this section, the authors also present results of the frequency stability over time and the lower limit of mass resolution. Section 4 is devoted to the synchronization process itself, computing the Arnold tongue for both synchronization methods and including a discussion of the mass sensing limitations working synchronously. Finally, Section 4 also presents a short discussion of the possible implications of the synchronization phenomena with noise. 

## 2. Materials and Methods 

### 2.1. CMOS-MEMS System Design and Fabrication

**Figure 1:**
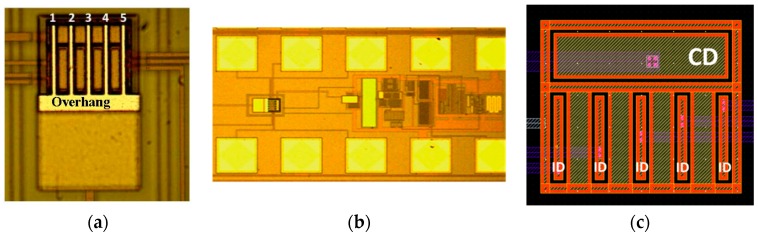
(**a**) Optical image of the five released cantilevers (26 μm long, 1.45 μm wide, 0.925 μm thick) in which the overhang can be seen (6 μm wide, 0.925 μm thick). The five cantilevers are numbered to reference them in the main text; (**b**) Optical image of the whole system, including the cantilever array and CMOS transimpedance amplifier; (**c**) Image of the layout of the drivers with the individual drivers (ID) and common driver (CD) and their access. CD common driver is below the tip of the cantilevers and the ID individual drivers are between the middle and near-to-anchor part of the cantilevers.

The CMOS-MEMS system is fully integrated using Austria MicroSystems (AMS) 0.35 μm CMOS technology [20]. The whole system is shown in Figure 1, which comprises five cantilevers electrostatically sensed and actuated out of plane and a CMOS transimpedance amplification circuitry [21]. The cantilevers were fabricated using the Metal 4 layer of AMS technology, clamped together, and mechanically connected through an overhang (see Figure 1a). Each cantilever is 26 μm long, 1.45 μm wide and 0.925 μm thick; the overhang is 6 μm wide and has the same thickness as the cantilevers. Read-out drivers were fabricated using the Metal 3 layer of AMS technology, 1 μm below Metal 4 layer, which will be the gap distance between cantilevers and read-out or actuation drivers. There is one common read-out driver (CD) connected to the transimpedance amplifier to sense the five cantilevers at the same time. This common driver is placed at the tip of the cantilevers (see Figure 1c). There is also one individual driver (ID) for each cantilever, connected directly to our characterization setup without amplification, which allows the individual actuation/sensing. There is a surrounding shield for all drivers connected to the ground, which is made of a Metal 3 layer, in order to minimize the external noise (frame wrapping common and individual drivers, see Figure 1c). 

The definition of the MEMS cantilevers is completely done in the AMS foundry. An in-house releasing process of the MEMS movable parts is done on the received bare chips, which require only one post-processing step in order to eliminate the sacrificial oxide surrounding the structural metal parts of the MEMS resonator (in our case, the five cantilevers). This post-processing step consists of immersing the chip in buffered hydrofluoric acid (BHF) and then rinsing it with deionized water and isopropyl alcohol to avoid stiction between the cantilevers. The engraving time has to be carefully calibrated to avoid undesirable under-etching, taking into account that the deeper the layer, the longer the engraving time. The chips are protected by the CMOS passivation layer and, since a window is opened in the passivation layer over the multicantilever system, obtaining in this way a pool-like structure, BHF engraves only the chip zones where the multicantilever system is, releasing the MEMS system.

The releasing post-process step and the dimension tolerances from the CMOS technology used add some sort of anisotropy to the modal frequencies of the cantilevers (providing a different resonant frequency for each of the cantilevers). In fact, the materials around the system and the pool-like structure break the symmetric surrounding of the five cantilevers under the BHF releasing process, varying the dimensions of each cantilever in relation to its neighbors and, therefore, producing a dispersion of modal frequencies. This inconvenience could be overcome by tuning the resonance frequency by applying DC voltage at the individual drivers, taking advantage of the spring softening effect. 

### 2.2. CMOS-MEMS System Electrical Characterization

**Figure 2:**
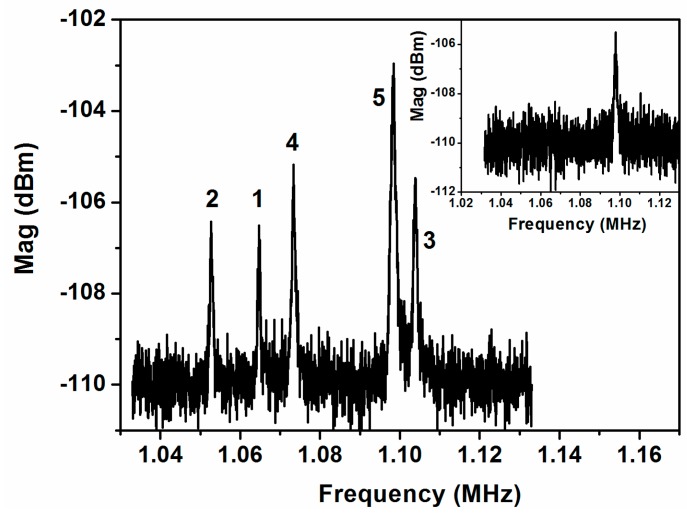
Thermomechanical noise measured at the common driver applying a DC voltage of 24 V at the system anchor. We flag the five peaks to identify each one with its corresponding cantilever (shown in Figure 1a). Peak number 5 is enhanced by the parasitic noise peak. Inset: parasitic noise peak measured at 0 V of DC voltage.

All the measures have been performed in vacuum conditions. To characterize the natural resonance frequency of each cantilever, we have measured the thermomechanical noise at the common driver, applying a DC voltage of 24 V at the system anchor and using Keysight N9030A signal analyzer with 10 Hz of IF bandwidth. Five different frequency peaks corresponding to the five cantilever MEMS resonators have been obtained (see Figure 2) and an additional one is due to external noise. Applying a DC voltage at each individual driver (see Figure 1), we can use the spring softening effect to relate each peak to the corresponding cantilever. Peak number 5 (Figure 2) is a sum of the peak corresponding to cantilever number five and the parasitic external noise (demonstrated by measuring the thermomechanical noise at 0 V of DC effective voltage), resulting in a higher magnitude peak compared with the others. 

**Figure 3:**
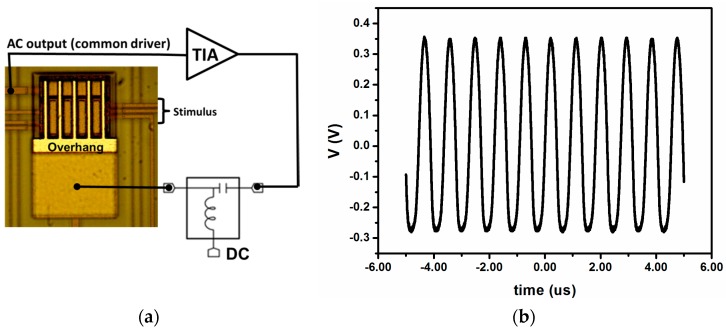
(**a**) Schematic of the closed-loop setup, showing the capability of additional stimuli at individual drivers and an optical image of the five-cantilever array; (**b**) Time response of the closed-loop oscillation (applying a DC voltage of 24 V).

In order to characterize the frequency stability of the system, we performed closed-loop measurement, taking the signal from the transimpedance amplifier (TIA) connected to the common driver, adding DC voltage of 24 V through a bias-tee, and driving this signal to the anchor of the system (see Figure 3a). In this configuration, the system is self-oscillating (see Figure 3b). The frequency was acquired using a Hewlett Packard 53131A frequency counter (Keisight Technologies, Santa Rosa, CA, USA).

**Figure 4:**
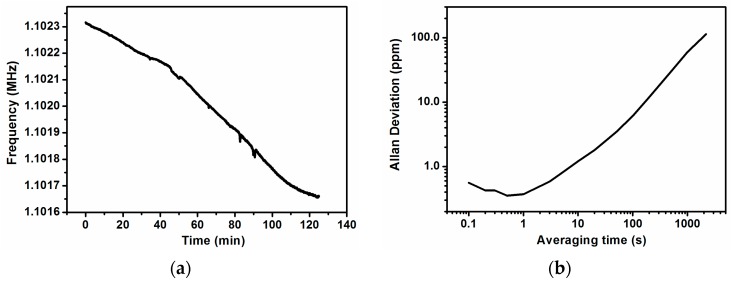
(**a**) Frequency stability measured in closed-loop configuration for 120 min. There is a clear long term drift; (**b**) Allan deviation in ppm for the closed-loop measurements with Vdc = 24 V and taking measures every 0.1 s. The minimum Allan deviation is near 1 s of averaging time.

We can see in Figure 4a the stability of the frequency of oscillation, taking measurements for two hours (each measure is taken every 0.1 s), in which we can appreciate a long time drift (corresponding to a linear drift of 0.1 Hz/s). The system auto-oscillates at the frequency corresponding to peak number 3 of Figure 2. The corresponding Allan Deviation from time measurements every 0.1 s, shown in Figure 4b, has a value below 1 ppm at 1 s of averaging time.

### 2.3. CMOS-MEMS System Mass Resolution

Following the same procedure as [21], we can calculate the mass sensitivity using the standard Equation (1), where *m_eff_* is the effective mass of the system, *f*_0_ is the resonance frequency in auto-oscillation mode, and ∆*f* is the dispersion of frequencies, ∆*f* = *f*_0_·σ, where σ is the Allan Deviation for the specific averaging time. For our system under self-oscillation conditions, *f*_0_ = 1.102 MHz, *m_eff_* = 1.1 × 10^−10^ kg and the frequency dispersion is ∆*f* = 0.3 Hz, which corresponds to σ = 3.5 × 10^−7^ (at an averaging time of 1 s). Consequently, the minimum achievable mass detection, or mass resolution, is 60 ag (attograms).

(1) $$\Delta m=\frac{2meff}{f_{0}}\Delta f$$

## 3. Characterization of the Cantilever Array under Synchronization

As is reported in the works mentioned above (see [13], for example), one of the special features of the synchronized state is the enhanced frequency stability. In our CMOS-MEMS system, the frequency dispersion between the individual cantilevers due to the post-process and dimensions tolerance of the fabrication is too high to allow for natural synchronization. For natural synchronization it is mandatory to have a commensurable relationship *n*:*m*, where *n* and *m* are integers, between resonance frequencies of the oscillators to be synchronized [3]. Measuring the closed-loop oscillation with our five-cantilever array CMOS MEMS system, we are not able to observe synchronization. Despite the fact that the system oscillates (at closed-loop configuration) at one individual frequency (and not five), the fluctuations of it and the long time drift are high enough to discard a synchronized state. Due to this inconvenience, we changed our measurement procedure. 

We used the closed-loop configuration for the following measurements, in order to have a self-sustained system (a system that oscillates taking energy from a source, in our case, a DC voltage) [3]. We achieved synchronization by applying an external electrical force (stimulation) through a sinusoidal signal (from the function generator, Agilent 81150A, (Keisight Technologies, Santa Rosa, CA, USA) to an individual driver (ID, see Figure 1). We used two of the five cantilevers, numbers 2 and 4 (see Figure 1a), as candidates for this external forced synchronization.

We used two methods; one is based on applying an external force to one of the individual drivers using the same frequency as the modal frequency of the corresponding individual cantilever. In this case the external force induces the cantilevers’ synchronization (see Section 3.1). The other method is based on applying this force, also on an individual driver, but with the same frequency as the self-oscillation state has. In this case the external force synchronizes with our self-sustained system (see Section 3.2). A discussion of these two methods of external excitation is presented in Section 4. 

### 3.1. Using an External Force Applied to One of the Individual Cantilevers, at the Same Frequency as the Modal Frequency of the Corresponding Cantilever

**Figure 5:**
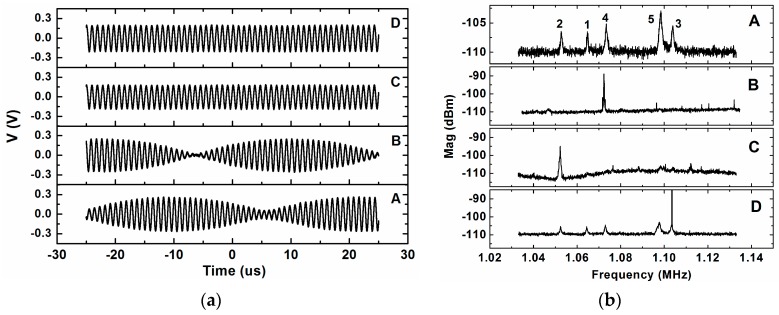
(**a**) Time response signal of the closed-loop self-oscillation of the system with stimulation on cantilever number 4 with DC voltage of 18 V and using different amplitudes peak-to-peak: **A**, 350 mV; **B**, 450 mV; **C**, 500 mV; **D**, 600 mV; (**b**) Thermomechanical noise plus excitation with DC voltage of 24 V in open-loop configuration: **A**, without excitation; **B**, excitation on cantilever number 4 at its modal frequency with 650 mV of amplitude, where we can see that peak number 4 stands above the others; **C**, excitation on cantilever number 2 at its modal frequency with 650 mV of amplitude, where we can see that peak number 2 stands above the others; **D**, excitation on cantilever number 2 at the modal frequency of cantilever number 3, where we can see that the five peaks remain and a large and sharp peak appears as number 3.

The external force is applied (as we said before) on one individual driver (ID) at the same frequency as the corresponding cantilever modal frequency with different amplitudes. The time-dependent signal of the system response is acquired, in a closed-loop configuration, at the common driver, for an applied DC voltage of 18 V and for different stimulation amplitudes. In Figure 5a we can see the time domain response for different stimulation amplitudes at the cantilever number 4 (and at its modal frequency) acquired with the oscilloscope Agilent DSC-X 3054A. We have a sum of two signals with different frequencies resulting in a pulsed time-dependent signal, until the amplitude reaches 500 mV (peak-to-peak), where the signal stabilizes at a single frequency, which corresponds to the modal frequency of the stimulated cantilever (number 4). If we analyze the signal (using Fourier transform) at 500 mV of excitation amplitude, we obtain a single peak at the modal frequency of the stimulated cantilever, which is a signature of synchronized state. On the other hand, if we analyze the pulsed signal (using Fourier transform) we conclude that the pulse is due to the sum of two signals, one at 1.076 MHz, corresponding to the modal frequency of cantilever number 4 (frequency of stimulation), and another signal with a frequency of 1.108 MHz, corresponding to the self-oscillation frequency of the system without additional stimuli (both applying a DC voltage of 18 V). Based on these measurements, we decided to use a stimulation of 650 mV of amplitude to ensure, as much as possible, a single value of the frequency for the read-out signal. Figure 5b, in an open-loop configuration, shows the effect on thermomechanical noise when applying an excitation to the corresponding cantilever (row A corresponds to the pure thermomechanical noise, without excitation; row B corresponds to the thermomechanical noise plus excitation of cantilever number 4 and rows C and D to cantilever number 2). 

Figure 5b shows that, when we apply an excitation over an individual cantilever at its modal frequency (Figure 5b, rows B and C), the peaks corresponding to the other cantilevers disappear and only the one corresponding to the excited cantilever remains; moreover, this peak has higher power than in the case without excitation (for example, we can compare peak number 2 of rows A and C or peak number 4 in rows A and B of Figure 5b). We deduce that, in this case, the system is synchronized and oscillates at the modal frequency of the excited cantilever. When the excitation is applied to the cantilever but at a different frequency, the five peaks remain and no traces of synchronization appear. An example of this is shown in row D on Figure 5b, where excitation is applied over cantilever number 2 but at the frequency corresponding to cantilever 3; we can see the five individual peaks as in row A of Figure 5b (except a sharper peak coming from the excitation itself), contrary to row C on Figure 5b, in which only peak number 2 remains. 

**Figure 6:**
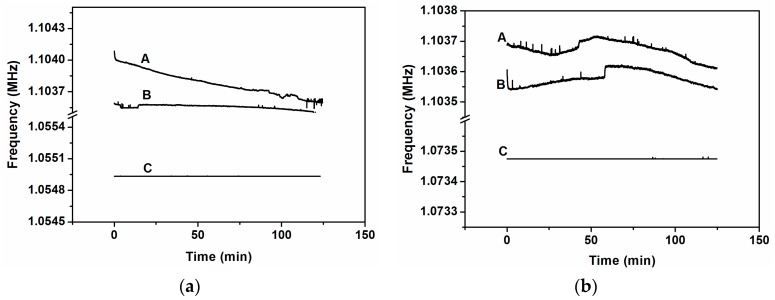
(**a**) Closed-loop frequencies for stimulus at cantilever number 2 (modal frequency, *f*_02_ = 1.0549 MHz) with different frequencies: **A**, at 1.06 MHz (above *f*_02_); **B**, at 1.04 MHz (below *f*_02_); **C**, at 1.0549 MHz (exactly at *f*_02_); (**b**) Closed-loop frequencies for stimulus at cantilever number 4 (modal frequency, *f*_04_ = 1.07348 MHz) with different frequencies: **A**, at 1.08 MHz (above *f*_04_); **B**, at 1.06 MHz (below *f*_04_); **C**, at 1.07348 MHz (exactly at *f*_04_).

**Figure 7:**
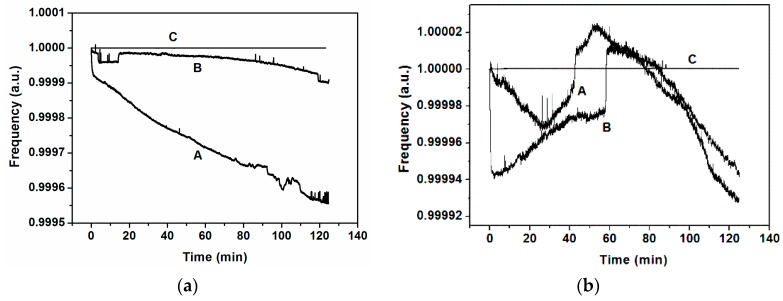
(**a**) Closed-loop frequency scaled at the starting point for the case of stimulus at cantilever number 2 with different frequencies: **A**, at 1.06 MHz; **B**, at 1.04 MHz; **C**, at 1.0549 MHz; (**b**) Closed-loop frequency scaled at the starting point for the case of stimulus at cantilever number 4 with different frequencies: **A**, at 1.08 MHz; **B**, at 1.06 MHz; **C**, at 1.07348 MHz.

Following the same steps mentioned in Section 2 but now using stimulation, we measured the frequency stability of the system for 120 min. The procedure was as follows: we stimulated one cantilever, number 2 or number 4, applying the signal at its own individual driver and at its own modal frequency; the closed-loop was done as previously, taking the signal from the transimpedance amplifier connected to the common driver and driving this signal to the anchor of the system adding, through a bias-tee, a DC voltage of 24 V. We observed that, when the stimulation is at the modal frequency of the corresponding stimulated cantilever, the whole system oscillates at this frequency; otherwise the system oscillates at the frequency of cantilever number three (as without stimulation), as we can see in Figure 6 for both cantilevers. To discard the possible effect of the stimulus over the common driver, which could produce a read-out signal directly from the signal generator instead of the cantilevers system, we performed stimulus at the modal frequency and at two frequencies near the modal, one above and another below it. Only when the stimulus is at the modal frequency does the read-out signal have this frequency; otherwise, it has the frequency of the closed-loop without stimulus. Added to this fact, there is another and more important effect: the high frequency stabilization obtained when the stimulus is at the modal frequency of the corresponding cantilever. For instance, from Figure 6a, the time frequency stability corresponding to case A is around 0.05 Hz/s; for case B it is 0.005 Hz/s and for case C it is only 5 × 10^−7^ Hz/s. In Figure 7, we have adjusted the graphics of Figure 6 to highlight this effect, scaling the frequencies to the frequency of the starting point. When the stimulus is at a different frequency than the modal, the frequency stabilization does not occur. 

**Figure 8:**
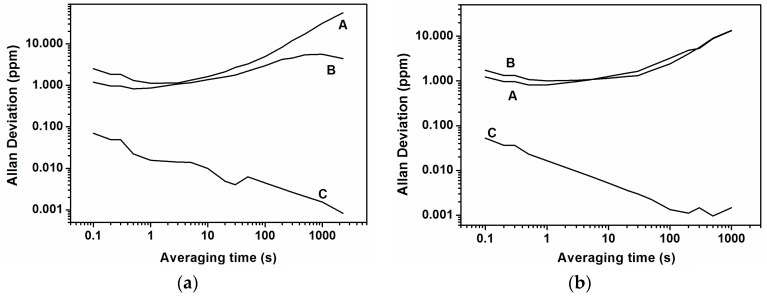
(**a**) Allan deviation in ppm stimulating the cantilever number 2, taking measures every 0.1 s and for stimulus frequencies of: **A**, at 1.06 MHz; **B**, at 1.04 MHz; **C**, at 1.0549 MHz; (**b**) Allan deviation in ppm stimulating the cantilever number 4, taking measures every 0.1 s and for stimulus frequencies of: **A**, at 1.06 MHz; **B**, at 1.08 MHz; **C**, at 1.07348 MHz.

Once we measured the closed-loop frequency with an applied stimulus, the next step was to evaluate the frequency stability calculating the Allan deviation (with measures every 0.1 s) under the same conditions. We can see in Figure 8 that cases A and B present similar behavior to that found without synchronization (Figure 4b), and that case C improves the Allan deviation by almost two orders of magnitude (@ 1 s of averaging time). Due to the fact that the stability of the frequency is very good for a long time, i.e., there is no frequency drift (see case C in Figure 6 and Figure 7), the Allan deviation decreases continuously and, in our measurement time window, we are not able to observe the Allan deviation limit due to the Flicker noise (Flicker floor). In addition to that and further supporting our claim that there is no direct effect between stimulus and read-out, the Allan deviation for stimulus at different frequencies is at the same order of magnitude as for the self-oscillation without stimulus (see Figure 8).

From Figure 8a, the frequency stability at an averaging time of 1 s, for the case of the stimulus applied at cantilever number 2 (at its modal frequency), is ∆*f* = 0.016 Hz (σ = 1.5 × 10^−8^), more or less the same value as for the stimulus applied at cantilever number 4. Using Equation (1), for the stimulus at cantilever number 2 or 4, we achieve a minimum detectable mass of 3 ag. We can further decrease the final mass resolution using a higher averaging time. For instance, with an averaging time of 100 s, the frequency dispersion is ∆*f* = 0.0011 Hz, and consequently the minimum achievable mass is 0.2 ag. In summary, from this synchronization technique, using forced cantilevers with stimulation at their own modal frequency, we can achieve a minimum detectable mass 300 times lower than in the case without stimulus with a long averaging time. 

These minimum achievable masses are calculated using Equation (1). Due to the fact that we have a synchronized system through the presence of an external force, these minimum achievable masses have to be taken as a lower limit of achievable masses. See the discussion of this fact in Section 4.2.

### 3.2. Using an External Force Applied to One of the Individual Cantilevers at the Self-Oscillating Frequency of the System

The synchronization phenomenon is not restricted to an interaction between oscillators; it is possible to synchronize an external force with a self-sustained system through a weak interaction as well [3]. The previous sub-section shows that, applying an external force to the cantilevers (at their own individual driver), they can change the self-oscillation frequency to the modal frequency of the actuated cantilever; otherwise, no changes are measured. This is not proper synchronization with an external force.

**Figure 9:**
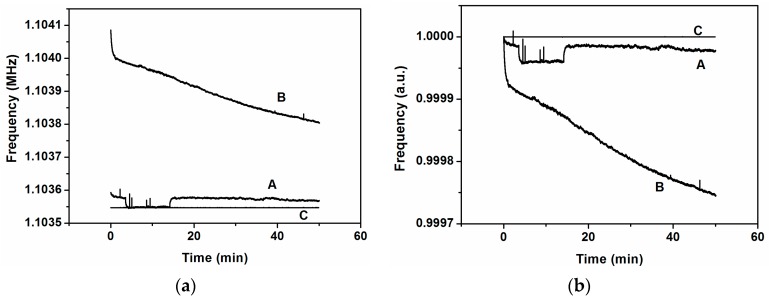
(**a**) Closed-loop frequency for stimulus at cantilever number 2 for different frequencies: **A**, at 1.04 MHz; **B**, at 1.06 MHz; **C**, at 1.103552 MHz; (**b**) Scaled frequencies by the starting point for the same cases than in (**a**).

To achieve synchronization with an external force, we proceed in a similar way to that used in the previous sub-section. We apply a stimulus to one of the individual drivers but, in this case, at the same frequency as the self-oscillation frequency, i.e., at the frequency of the peak number 3 (see Figure 2). We present the results in the case of stimulating the cantilever number 2 (although similar results were obtained with the stimulus applied to cantilever number 4). Figure 9 depicts the frequency evolution over time, showing stabilization only when the stimulus is at the same frequency as the self-oscillation (curves A and B in Figure 9a). For instance, from Figure 9a, the time frequency stability corresponding to case A is around 0.003 Hz/s; for case B it is 0.08 Hz/s and for case C it is 4.6 × 10^−8^ Hz/s. This fact is highlighted in Figure 9b, scaling the frequencies by the starting point. 

**Figure 10:**
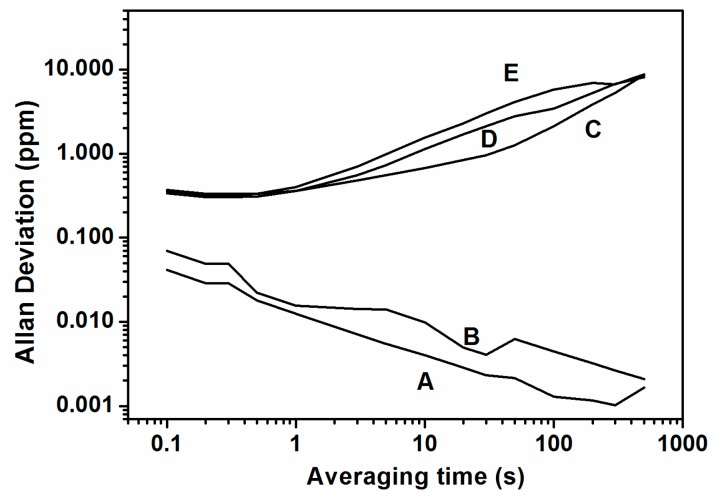
Allan deviation, exciting cantilever number 2 at different frequencies corresponding to the different peaks of Figure 2a: **A**, peak number 3 (1.10355 MHz); **B**, peak number 2 (1.05494 MHz); **C**, peak number 1 (1.06473 MHz); **D**, peak number 4 (1.0733 MHz); **E**, peak number 5 (1.09819 MHz).

In Figure 10 we can see the Allan deviation acting over cantilever number 2 with different frequencies corresponding to the modal frequency of each of the five cantilevers. When the frequency of the stimulus takes the same value as the self-oscillation frequency or the modal frequency of the corresponding cantilever, the Allan deviation has the minimum value (curves A and B of Figure 10). If we measure the frequency dispersion for 1 s of averaging time for the stimulation at the self-oscillation frequency (curve A in Figure 10), we obtain ∆*f* = 0.013 Hz, and using Equation (1) we obtain a minimum detectable mass of 2.6 ag. As we said previously, the minimum detectable mass calculated here (using Equation (1)) is a lower limit (see Section 4.2 for more details)**.**

Table 1 summarizes the frequency dispersion considering the different synchronization techniques. Note that the synchronization allows a 23-fold increase in the frequency dispersion for 1 s of averaging time.

## 4. Discussion

### 4.1. Considerations about Synchronization Using an External Force 

In this work, we present two ways of synchronizing the cantilevers: (a) exciting one of the cantilevers at its modal frequency and (b) exciting it at the self-oscillation frequency (the frequency in which the system oscillates without external stimulus on cantilevers). In both schemes we achieve the synchronization state using an external force. 

**Figure 11:**
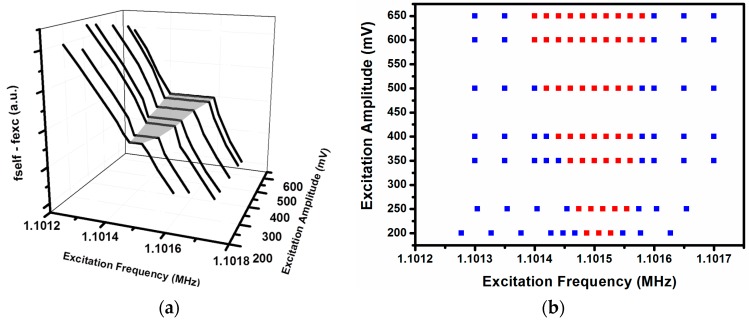
(**a**) The Arnold tongue, corresponding to excitation at the same frequency as the self-sustained (method (**b**)). The shaded gray zone is the synchronized zone, which widens when the amplitude of excitation grows; (**b**) A 2D representation of the Arnold tongue, in which the red squares represent the minimum difference between self-oscillation frequency and excitation frequency.

We discuss in this section the role of the external force as an agent for promoting synchronization due to the possibility that this external force can be synchronized with the system. Normally, one of the ways to study the synchronization between a self-sustained system and an external force is through the so-called Arnold tongue [3]. The Arnold tongue shows the region of frequencies in which the system is synchronized with the external force by plotting ∆*F* versus *f*, where ∆*F* is the difference between the frequency of the self-sustained system in the presence of the external force and the frequency of the external force, and *f* is the frequency of the external force. When the synchronization is achieved, a plateau appears, centered at the frequency of the self-sustained system without the presence of the external force. This plateau is wider as higher is the power of the external force. A 3D plot of ∆*F* versus *f* and versus power of the external force is the Arnold tongue (see, for example, [10]). Our second method of excitation (method (b)) can be represented using the Arnold Tongue, as can be seen in Figure 11. Figure 11a shows the synchronized zone, a shaded grey zone, which is a plateau that widens with the increasing of the excitation power (excitation amplitude in the *y*-axis of Figure 11). Figure 11b is a 2D representation of the Arnold Tongue that clarifies the effect of the excitation power over the synchronized zone. Red squares represent synchronized states and blue squares unsynchronized ones.

**Figure 12:**
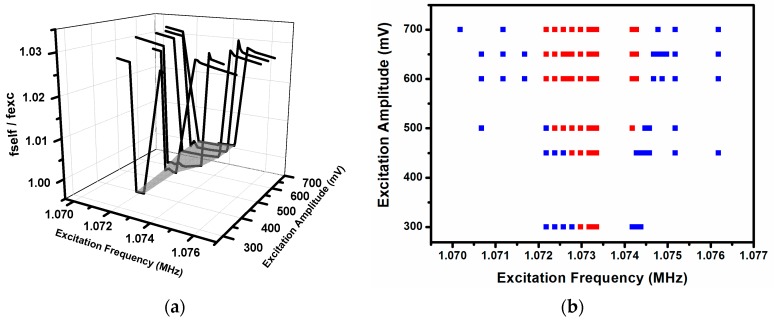
(**a**) Ratio between self-oscillation and excitation frequencies (*fself*/*fexc*) for different amplitudes of the excitation and 24 V DC of applied voltage. Here, the excitation is applied at cantilever number 4. The synchronized zone appears as a plateau for which the ratio *fself*/*fexc* takes the unit value (shaded gray zone); (**b**) A 2D representation of the previous figure, in which the red squares represent the minimum ratio of *fself*/*fexc* and the blue ones the maximum. We can see that the rage of excitation frequencies into the synchronized zone widens with the amplitude of the excitation.

Figure 12a shows a different representation of the Arnold tongue, adapted to our first method to achieve the synchronization (method (a)) at different amplitudes of the external force. To do this representation, we have done the measures using a frequency counter and applied the excitation (in Figure 12 the excitation is applied at cantilever number 4) at different frequencies. The *z*-axis represents the measure of the frequency counter. When we apply the external force with the same frequency as the modal frequency of the cantilever, the self-oscillation frequency changes from that corresponding with peak number 3 (at which frequency the system oscillates without external forcing; see Figure 2) to the modal frequency of the individual cantilever. In Figure 12a the synchronization zone appears as a plateau with the ratio between self-oscillation and excitation frequency equal to 1, and a different value for the out-of-synchronization zone (which corresponds to the ratio between the frequency of peak number 3 (self-oscillation frequency) and the frequency of the external force). The synchronization zone is centered at the modal frequency of cantilever number 4 and the range of excitation frequencies in which we have a synchronized state widens when the amplitude of excitation grows. Importantly, in the case of Figure 11a and Figure 12a, using 350 mV of excitation amplitude there is a thin synchronized zone. When we performed our first measures to obtain the time response signal (see Figure 5a), we obtained a modulated signal at 350 mV of excitation amplitude, which does not correspond to a synchronized state. This discrepancy is due to the fact that the time response signal measures were performed by applying 18 V DC at the closed-loop configuration, but in the last case the applied voltage was 24 V—more energy than in the first case, which caused the early appearance of the synchronized state. 

Figure 12b represents the widening of the synchronization zone in a more intuitive manner, capturing the synchronized zone with red squares and the non-synchronized zone with blue squares.

In the case of applying the external force with the same frequency at which the self-sustained system oscillates (the frequency of the self-oscillation in a closed-loop configuration), we conclude that the external force synchronizes with the system following the usual way [10] i.e., we are on the Arnold tongue plateau. This synchronization drives our system to a more stable oscillation frequency system than without the applied external force. We have to take into account that, when we use the closed-loop configuration, the frequency of oscillation of the system is unique, corresponding to the frequency of peak number 3 (see Figure 2); we do not have multivalued frequency as is measured in thermomechanical noise. It is important to emphasize that the external force is synchronized with the whole system.

When we apply an external force at the individual modal frequency of one of the five cantilevers, in our opinion, the way to achieve synchronization is not the same as in the previous case. In this configuration, the self-sustained system oscillates at a frequency (1.10355 MHz) that is very different to the modal frequency of one of the actuated cantilevers (for example, 1.07348 MHz for cantilever number 4). If we excite the system with an external force with the modal frequency of the corresponding actuated cantilever, we are exciting our system with a frequency far from the self-oscillation one, that is, we are out of the Arnold tongue plateau (showed in Figure 11a), which corresponds to synchronization to an external force and, consequently, a synchronization state between external force and self-sustained system should not be achieved. However, as we see, the whole system changes its self-oscillation frequency from 1.103 MHz to, for example, 1.073 MHz (if we excite cantilever number 4) and we obtain a synchronized system with an Arnold tongue (like the one depicted in Figure 12a), but shifted to the modal frequency of the excited cantilever. As we can see in Figure 2, the fabrication process produces a system with five cantilevers with enough differences between their modal frequencies to disallow the mutual synchronization. In our opinion, the role of the external force using the same frequency as the modal of the individual cantilever is helping the mutual synchronization between cantilevers. When the external force is applied at the modal frequency of the actuated cantilever, the whole system oscillates at this frequency (see Figure 6). This fact drives us to discard the direct influence of the force on the frequency of oscillation but to assert an indirect influence only when its frequency and the modal one of the cantilevers matches. 

### 4.2. Considerations about Mass Sensor Performance

Even though this synchronizable system could work with different purposes, we want to analyze it for mass sensing applications. We have introduced two ways to achieve synchronization with the purpose of achieving higher frequency stability. However, there are some considerations to be taken into account, directly related to the fact that, in synchronous operation, the system is locked with an external force. The Arnold tongue shows as a plateau the region in which the system frequency and the external force frequency are locked. It is important to state that, in order to use this synchronized system as a mass sensor measuring frequency change, the added mass should be able to shift the frequency by more than half of the width of the Arnold tongue plateau. Consequently, working with the minimum achievable power of the external force compatible with synchronization, i.e., the narrower zone of the Arnold tongue, is needed for mass sensing. 

In the case of an external force applied on one of the individual cantilevers, at the same frequency as the modal frequency of the corresponding cantilever, the spatial identification of the deposited mass over the five cantilevered array is achieved as is discussed below.

Imagine we have a system vibrating synchronously at the same frequency as the modal of one of the cantilevers, forced by an external force (the case described in Section 3.1). When we deposit mass over this cantilever, its modal frequency changes in direct relation to the amount of mass and then a mismatch between the modal frequency of the cantilever and the frequency of the external force appears. Consequently (if the intensity of the external force is not too high), the synchronization disappears (the system moves out of the Arnold tongue). Based on this fact, we will be able to identify which of the cantilevers has the added mass exciting sequentially each cantilever at its corresponding modal frequency, while deciding if the system works synchronously or not. The cantilever for which this procedure does not lead the system to work synchronously will be the cantilever where the mass has been deposited. This is an advantage due to the fact that we can perform a spatial detection of mass deposition. For instance, this method can be useful for profiling a flux of particles. Moreover, this method will allow the chemical identification of the deposited mass if we use a different and specific chemical functionalization of each cantilever in the array (for example, viruses caught by antigens sited at the corresponding cantilever, which will be spatially determined by losing the synchronization state). The disadvantage of this method of achieving synchronization is that, directly, we cannot measure the mass deposited because we cannot measure the shift of frequency (i.e., Equation (1) is not applicable in this case). To know the mass we have to perform another step. Centered at the identified cantilever (the cantilever with the added mass), we have to change the frequency of the external force until the system returns to a synchronous state. The difference between this new frequency and the previous one (the modal frequency of the cantilever without added mass) will determine the mass deposited at the cantilever. It is important to state that if we want to achieve the minimum detectable mass, we have to use the minimum external force to have the minimum range of synchronous state (the narrower zone of the Arnold tongue). This procedure requires a previous calibration of the system and two steps of excitation detection. 

### 4.3. Towards a Thermomechanical Noise Limit?

Recently there has been an exhaustive review of the frequency stability of micro and nanomechanical resonators [2]. This review states that the frequency stabilities of the NEMS resonators studied are far from the thermomechanical limit and none of them attain this limit. The authors discard the idea that this discrepancy between measurements and thermomechanical limit is due to the measurement system; rather, it originated in the mechanical domain of the device. The authors also disagree with the idea that the difference between the measured frequency fluctuations and the thermomechanical limit is due to temperature variations and another known mechanisms and conclude that there is a need for studying new microscopic mechanisms, which might be the origin of these discrepancies. According to this paper, the measured frequency fluctuation is on average two orders of magnitude (100-fold) greater than the thermomechanical frequency fluctuation limit.

We can evaluate in the same terms as [2] our CMOS-MEMS cantilevered array’s ability to compare stability with a synchronized operation mode and without. For our system the thermomechanical Allan deviation limit is computed to be 4.6 × 10^−9^ with 1 s averaging time (see the supplementary information in [2] for how to compute it). From Figure 10, the Allan deviation is 3.5 × 10^−7^ (@ τ = 1 s) without synchronized operation, which represents a factor 80x greater than the thermomechanical frequency stability in accordance with predictions and results in [2]. On the other hand, the Allan deviation becomes 1.3 × 10^−8^ (@ τ = 1 s) in a synchronized mode of operation, which represents an increase of only 2.6x in comparison with the equivalent thermomechanical limit, surpassing in this way the expected frequency stability for the majority of NEMS resonators. This opens the way to further push the limits for hig- performance mass sensors. Additionally, in our opinion, synchronized systems are revealed as a good strategy to attain the thermomechanical limit, and this fact must be taken into account to face the research concerning the new microscopic mechanism proposed by the authors in [2].

## 5. Conclusions

We present here a multicantilevered system with enhanced frequency stabilization of the self-oscillation frequency through synchronization. We have concluded that the frequency stability improves by around two orders of magnitude using synchronization phenomena. We present two ways to synchronize the cantilevers, both using an external force: one of them is to use this external force as a way to overcome the difficulties related to the dispersion of dimensions of the cantilevers due to the fabrication process, acting on an individual cantilever at its modal frequency and driving the whole system to oscillate at this frequency; the other is synchronizing the external force with the self-sustained system. For both synchronization methods we have presented the advantages and disadvantages using this system as a mass sensor. 

## Figures and Tables

**Table 1:** Frequency dispersion for different averaging times.

Measure Type	Frequency Dispersion at 1 s Averaging Time	Frequency Dispersion at 100 s Averaging Time
Without stimulation	0.3 Hz	6.8 Hz
Stimulation at cantilever number 2 at its modal frequency	0.016 Hz	0.004 Hz
Stimulation at cantilever number 4 at its modal frequency	0.017 Hz	0.0011 Hz
Stimulation at cantilever number 2 at the self-oscillation frequency	0.013 Hz	0.0013 Hz

## Abbreviations

The following abbreviations are used in this manuscript:

CMOS	Complementary Metal Oxide Semiconductor
M/NEMS	Micro/Nano ElectroMechanical Systems
TIA	TransImpedance Amplifier
